# Comparative Study on Electrochemical Treatment of Cyanide Wastewater

**DOI:** 10.3389/fchem.2021.598228

**Published:** 2021-03-19

**Authors:** Siming Lei, Yonghui Song

**Affiliations:** ^1^School of Metalhurgical Engineering, Xi'an University of Architecture and Technology, Xi'an, China; ^2^Key Laboratory of Gold and Resource of Shaanxi Province, Xi'an, China

**Keywords:** voltage, electrode material, three dimensional electrode, cyanide wastewater, coal based electrode

## Abstract

The electrochemical treatment of wastewater is widely used for cleaning due to its efficiency. In this paper, two-dimensional (2D) and three-dimensional (3D) electrochemical systems were used to treat cyanide wastewater. The effect of the applied voltage and the material of the main electrode on the removal of various ions and the characteristics of chemical reactions were mainly studied. The results show that the applied voltage was the key effect of the electrochemical treatment process. The removal of ions from the wastewater at 2 V is mainly due to the effect of electro adsorption and enrichment precipitation, while at 4 V, it is mainly due to anodization and cathodic deposition. The treatment effect of the 3D electrode system was significantly better than the 2D system. The 3D electrode system by used granular activated carbon as the particle electrode, with the carbon filled stainless mesh (CM) and coal based electrode (CB) as the main electrode, the treatment effect were better than main electrode of stainless steel mesh (M). The 3D system with CB as the main electrode had an applied voltage of 4 V, a treatment time of 5 h, plate spacing of 10 mm, and the dosage of activated carbon particles was 2 g. The removal rates of CN_T_, Cu, Zn, CN^−^, and SCN^−^ were 94.14, 94.53, 98.14, 98.55, and 93.13%, respectively. The main reaction in anode was the electroly oxidation of CN^−^ and SCN^−^, while the electrolytic deposition of Cu, Zn, and other metal ions in the cathode surface. There were not only adsorption and electric adsorption of various ions, but also an electrolytic deposition reaction of Cu, Zn, and other metal ions on the surface of the activated carbon particle electrode. During the electrochemical reaction, the concentration of hydrogen ions near the anode increases locally, which produces the precipitation of CuSCN, Cu_2_Fe(CN)_6_, and Zn_2_Fe(CN)_6_, etc. in the solution, which are helpful for the removal of cyanide and heavy metal ions in cyanide wastewater.

## Introduction

Cyanide wastewater treatment has been the subject of research due to the difficulty of treating its complex composition, which contains a large number of free cyanide and copper, zinc, iron metal cyanide complex ions. Three-dimensional (3D) electrode electrochemical system treatment techniques are used to remove organic material and heavy metal ions from industrial wastewater of chemical, metallurgical, and material industries (Vedula et al., 2013). Due to the advantages of short mass transfer distance, high current efficiency, short treatment time, and low energy consumption, this method has gradually attracted the attention of researchers (Jia et al., 2013; Shilei et al., 2013). The 3D electrode system introduces a third electrode (particle electrodes) based on a two-dimensional (2D) electrode system. Under the action of the electric field, the particle electrodes and anode and cathode conduct electrochemical reactions together, resulting in higher removal efficiency (Jinzhi et al., 2015).

The 3D electrode system achieved results in terms of process and basic theory (Lei et al., 2013; Ting et al., 2018). At present, it is used for the electrochemical oxidation-reduction treatment of inorganic heavy metal ions including lead, zinc, copper, nickel, and chromium, and organic industrial wastewater from dyeing, papermaking, coking, and other industries. Xi et al. (2015) treated simulated acidic copper and nickel wastewater in the laboratory by electrodeposition, under optimal process conditions. The ion deposition rates of copper and nickel can reach 99.88 and 85.21%, which indicates the feasibility of using electrodeposition technology to treat polymetallic wastewater. Han and Zhenshan (2013) used activated carbon as particle electrodes in a 3D electrode system to treat wastewater that contained zinc. The results showed that with the voltage of 6 V, the removal rate of zinc ions was increased by 48%, the average current efficiency was increased by 40%, and energy saving was 70% compared with the 2D electrode system. Hui (2016) used a self-made coal based electrode material as an anode and cathode to treat cyanide wastewater. It was found that when the voltage was 2 V and the time was 5 h, the removal rates of CN_T_, Cu, Zn, and SCN^−^ in the solution were 75.17, 88.48, 29.51, and 47.57%, respectively. To examine the effect of voltage on the treatment of cyanide wastewater with a 3D electrode, Yao et al. (2017) used self-made coal based electrode material as an anode and cathode, and commercial activated carbon as particle electrodes to treat cyanide gold extraction wastewater. It was found that the voltage has an obvious influence on wastewater treatment. Electrolytic deposition played an important role in the 3D electrode system when the voltage was 4 V, time 5 h, and the electrode distance was 10 mm. The removal rates of CN_T_, Cu, Zn, SCN^−^, and CN^−^ in wastewater were 93.94, 95.2, 97.23, 99.38, and 94.93%, respectively.

The above studies show that the electrochemical treatment of cyanide wastewater with 2D/3D systems has a good treatment effect, and the electrode is an important determinant worthy of further study.

In the present study used a stainless steel mesh filled with activated carbon electrode (CM) coal-based electrode (CB) and stainless steel mesh (M) electrode. Commercial activated carbon particles were used as the particle electrode. The electrochemical treatment of cyanide wastewater by 2D and 3D systems and the effects of electrode materials and applied voltage on the removal law of various ions in cyanide wastewater and its mechanism was studied.

## Experimental

### Materials

The 304 stainless steel mesh (M) used in the experiment was from a steel product factory in Jiangsu Province. The filler and the particle electrode were commercial coconut-shell activated carbon. The low rank pulverized coal and liquefaction residue was from a coal chemical factory in Yulin, Shaanxi Province. The cyanide wastewater was from a gold smelter in Henan Province, and its main composition is shown in Table 1, which belongs to high concentration cyanide containing wastewater (Acheampong et al., 2010). Analytical grade reagents and deionized water were used.

**Table 1:** Main components of cyanide wastewater^a^ used in the experiment/mg·L^−^^1^.

**Components**	**CN_**T**_**	**CN^**−**^**	**Cu^**2+**^**	**Zn^**2+**^**	**SCN^**−**^**
Content	1872.6	383.4	593.2	468.8	262.15

a *pH = 7.30*.

### Experimental Procedures

Fifty milliliter cyanide wastewater was accurately measured taken into the electrolytic cell. The electrode plates were, respectively, connected with the positive and negative electrodes of the DC power supply. The plates were connected with wires plugged into the solution in parallel with 10 mm intervals. The experiment under different applied voltages, for a treatment time of 5 h, and the dosage of particle activated carbon in the 3D system was 2 g. At the end of the experiment, the solution was treated by liquid-solid separation, and several ions concentrations were regularly analyzed. The precipitates and plates were repeatedly washed with distilled water until pH 7 was reached, and then dried for analysis and characterization. The connection diagram of the experimental device was shown in Yao et al. (2017). In the experiment, the stainless steel mesh (M) used the 304 stainless steel (pore diameter ≤ 30 mesh), the activated carbon filled stainless steel mesh electrode (CM) used the stainless steel mesh as a framework (30 × 30 × 2 mm), and 2 g commercial activated carbon (had secondary activation by nitric acid) as filler. The preparation of the self-made coal based electrode (CB) followed a method previously described (Yonghui et al., 2016).

### Analytical Procedures

The surface morphology of the electrode material and the composition of the loading material were analyzed by JMS-6390Lv Scanning Electron Microscope (with energy-dispersive IE300X). The composition of the precipitations was analyzed by X-ray Diffractometer (D8 ADVANCE A25). The concentration of CN_T_ and CN^−^ in wastewater were analyzed by chemical titration, the concentration of Cu and Zn were analyzed by AA-1800H Atomic Absorption Spectrometry, and the thiocyanate ion was determined by 723PC Spectrophotometer.

## Results and Discussion

### Removal Rate of Various Ions at 2D System

**Figure 1:**
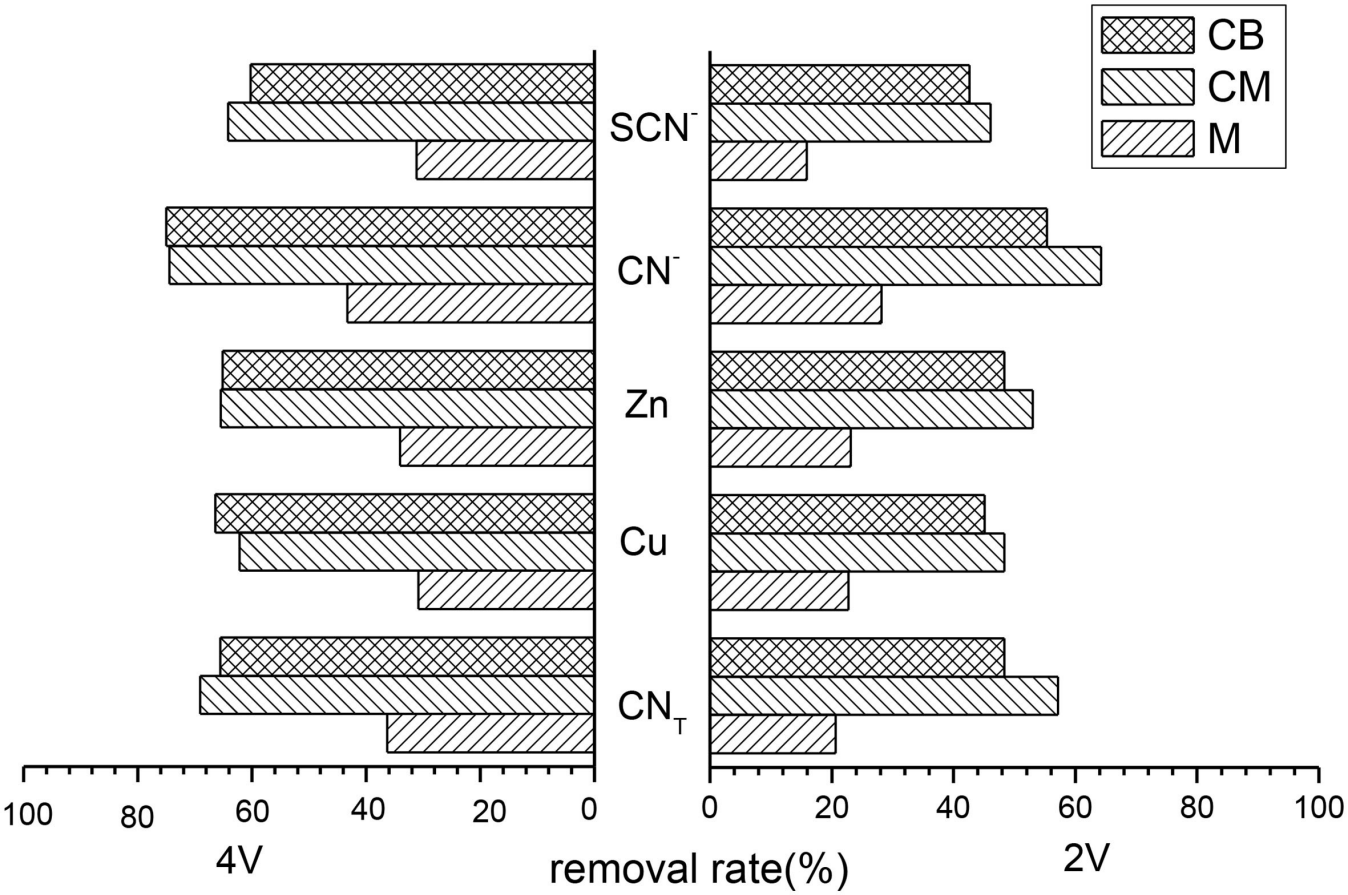
Removal rate of various ions with different main electrodes at 2D system (pH = 9, time = 5 h, voltage = 2 V or 4 V).

Treatment cyanide wastewater used the main electrodes of M, CM, and CB, respectively, under 2 and 4 V conditions by the 2D system. The removal rates of several main ions were shown in Figure 1. It can be seen from the results that the removal rates of ions for the three kinds of main electrodes were relatively low at 2 V, basically no more than 60%. While the removal rates of CN_T_, Cu, Zn, CN^−^, SCN^−^ at 4 V were all about 20% higher than those under 2 V. In addition, the treatment effect of the CM electrode was better than the CB electrode and M electrode, but the overall treatment effect was not good. When the main electrode was M, the removal rate of ions in 2 V was generally low and the highest removal rate was only 28.14% (CN^−^). The removal rate of all ions was greatly improved at 4 V, but only maintained levels at about 35%. This may be due to the small contact area between the electrode and wastewater. When CM and CB were used as the main electrodes, the removal rates of each ion in the 2D system reached more than 62% under 4 V conditions, and the removal rates were significantly improved. When the main electrode was CM, the removal rates of CN_T_, Cu, Zn, CN^−^, and SCN^−^ were 69.11, 62.17, 65.45, 74.43, and 64.14%, respectively.

### Removal Rate of Various Ions at 3D System

**Figure 2:**
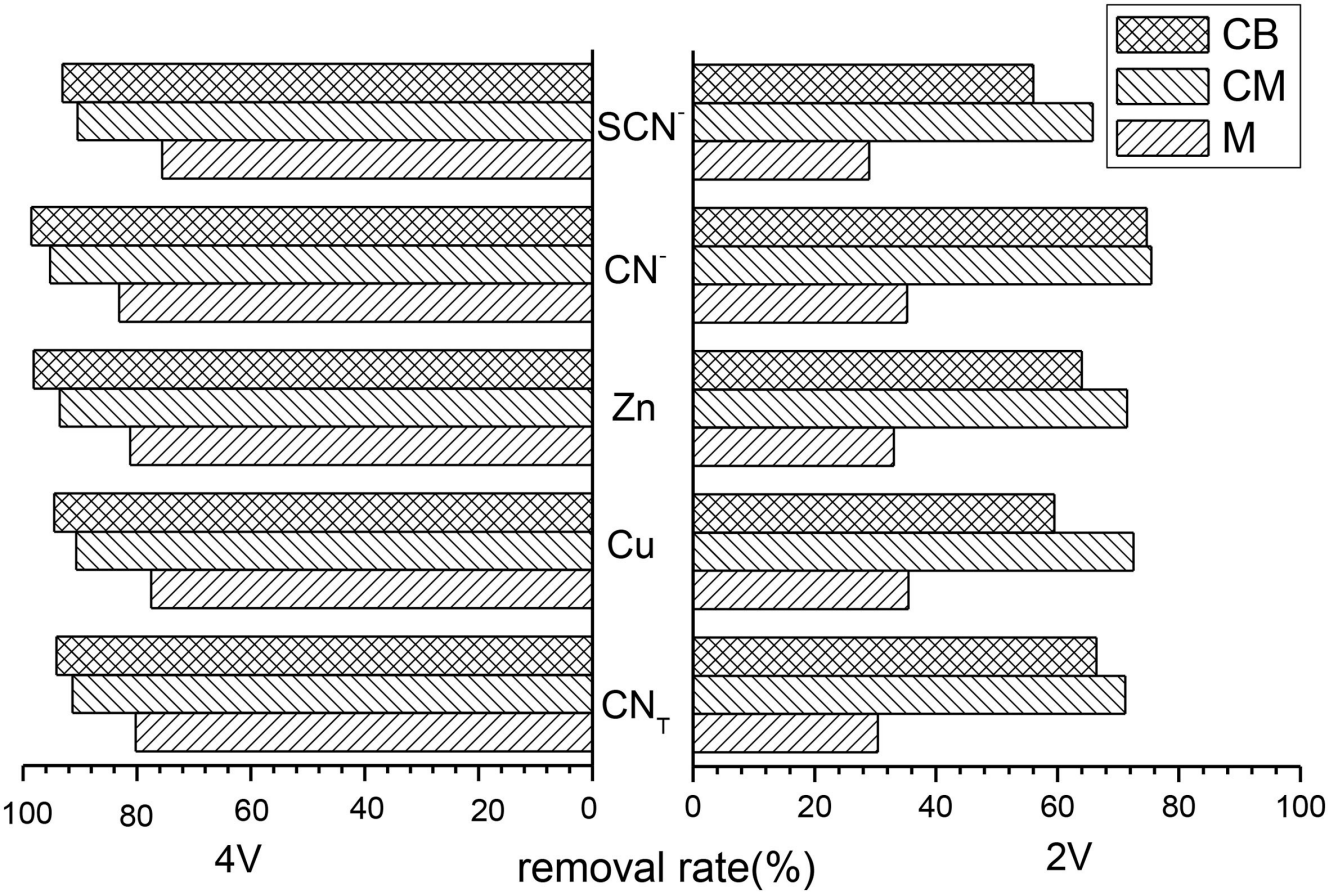
Removal rate of various ions with different main electrodes at 3D system (pH = 9, time = 5 h, dosage of activated carbon = 2 g, voltage = 2 V or 4 V).

Treatment cyanide wastewater in the 3D system used various main electrodes under the conditions of 2 and 4 V. The removal rate of several main ions is shown in Figure 2, which shows that the removal rates of ions in the three main electrodes were not high under 2 V conditions, especially the M electrode. The removal rates of each ions were only about 30% but were significantly improved under 4 V conditions. When CM and CB as the main electrodes, the removal rates of each ions in the 3D system under 4 V conditions were all above 90%. When the main electrode was CB, the removal rates of CN_T_, Cu, Zn, CN^−^, and SCN^−^ were 94.14, 94.53, 98.14, 98.55, and 93.13%, respectively.

Compared with the 3D system, the 2D system had a low treatment efficiency and was similar to the reaction process of the main plate with the 3D system. Therefore, the SEM-EDS analysis of the main plates and particle electrodes in this paper focused on the 3D system.

### SEM-EDS Spectrum Analysis of Anode

**Figure 3:**
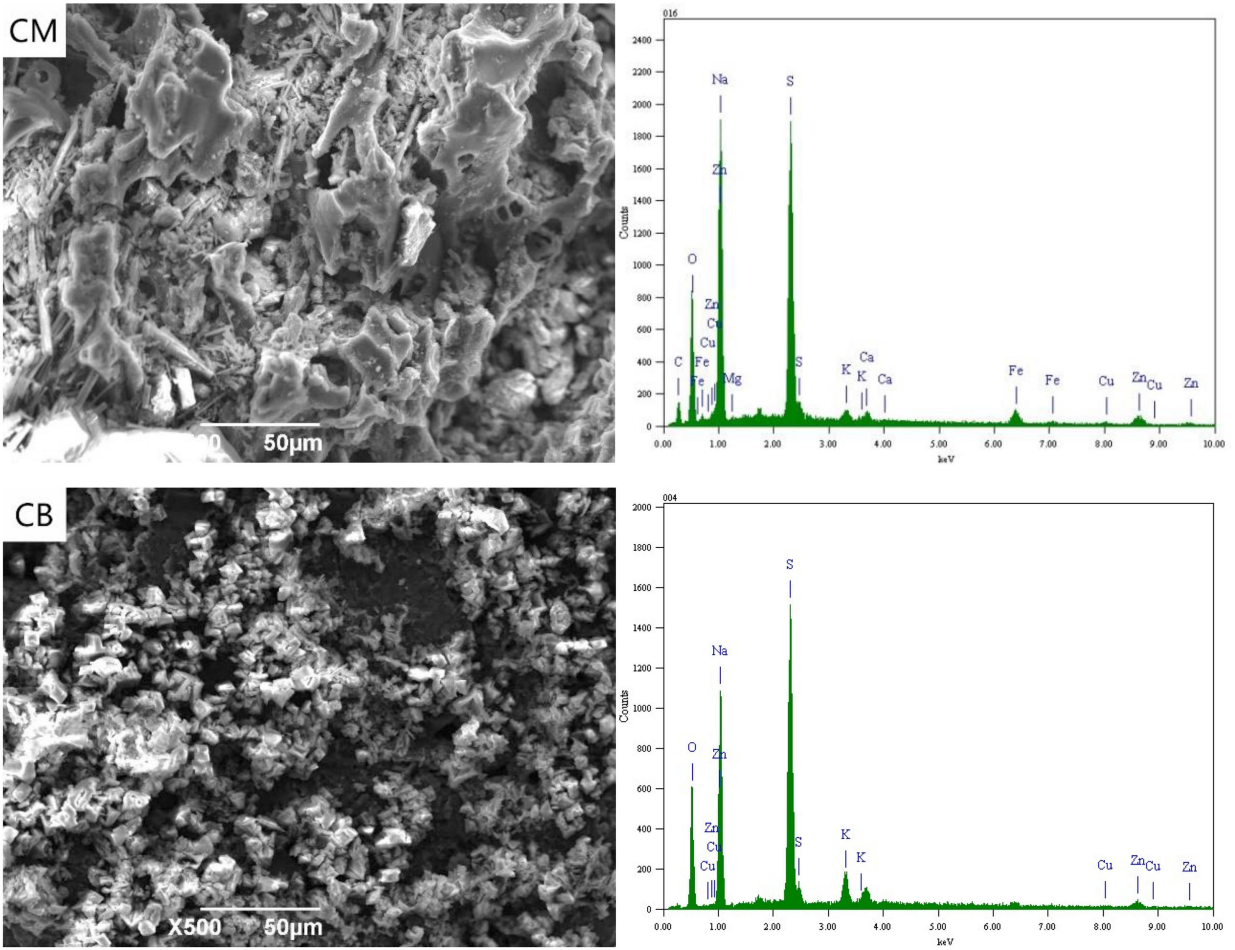
SEM-EDS spectrum of anode under 2 V at 3D system.

Figure 3 shows the SEM-EDS spectra of CM and CB anodes under the applied voltage of 2 V. It can be seen from the figure that the electrodes corroded after use. This was likely caused by the consumption of carbon in the reaction process and the erosion of the electrode sheet during the stirring process. Simultaneously, there were white attachments on the surface of the two kinds of anodes. The energy spectrum analysis results show that these substances were complex ions of Cu, Zn, Fe, and other anions. Besides the electrode plate's adsorption effect, the anions that were removed from the solution depend on the directional migration and adsorption on the anode surface under the electric field. The energy spectrum shows that there was a certain amount of Fe in the CM anode, which may be caused by the strong adsorption properties of activated carbon. The cyanide from wastewater and the iron in the stainless steel mesh produces ferricyanide preferential adsorption on the activated carbon.

When the voltage was 2 V, gas was produced at the anode. Oxygen evolution reaction occurs at the anode due to the electrode terminal voltage reaching the decomposition voltage of water. White precipitate appears near the anode, which was due to the high concentration of H^+^ and resulted in the hydrolysis of OH^−^ near the anode. This was caused by the precipitate reaction of H^+^ with complex ions and SCN^−^ in the solution, mainly including CuSCN, CuCN, Zn(CN)_2_, Cu_2_Fe(CN)_6_, Zn_2_Fe(CN)_6_, Cu_3_Fe(CN)_6_·4H_2_O, Zn(OH)_2_, and Cu(OH)_2_. The removal of ions in wastewater at 2 V was thus caused by the interaction of electric adsorption and anodic precipitation.

**Figure 4:**
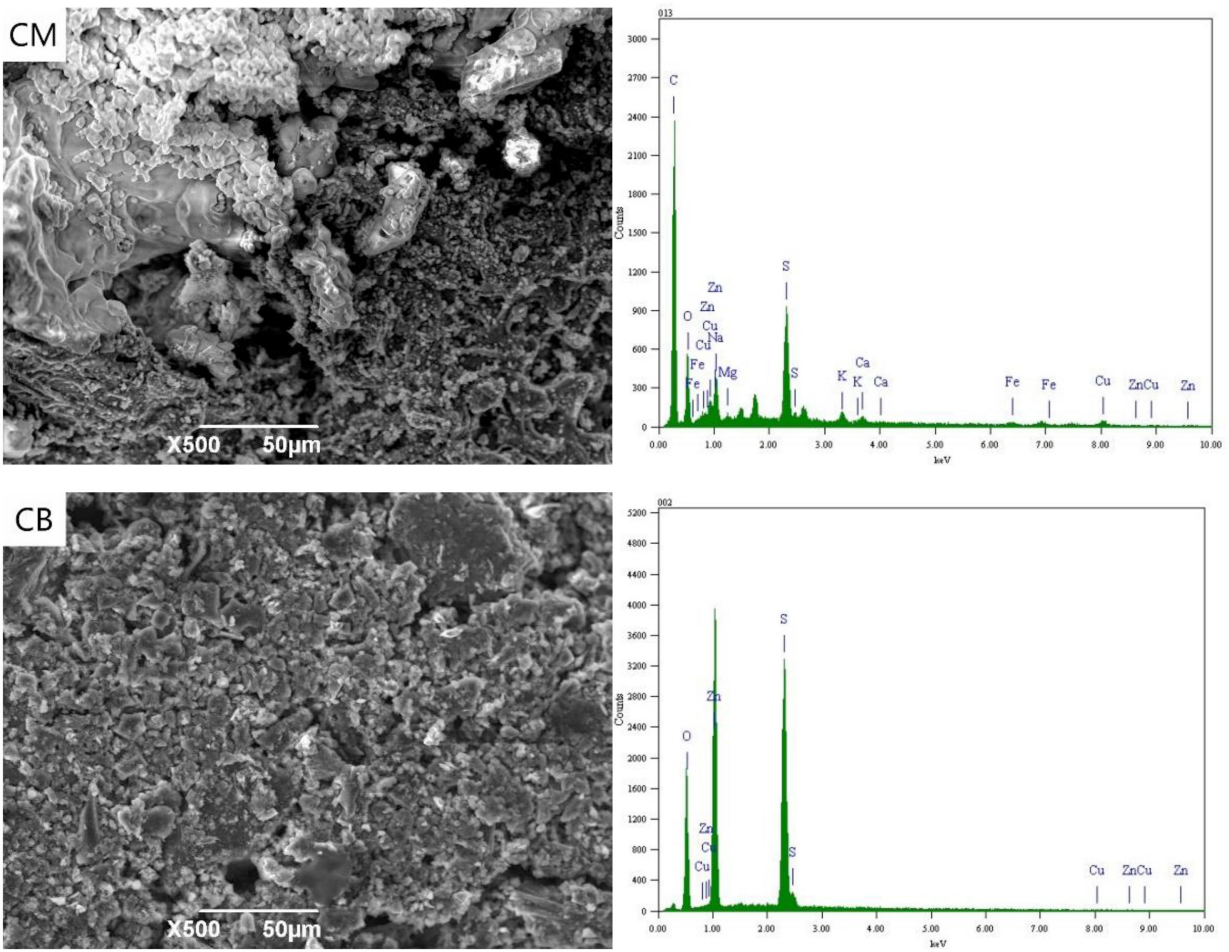
SEM-EDS spectrum of anode under 4 V at 3D system.

Figure 4 shows the SEM-EDS spectrum of the CM and CB anodes in the 3D system under the applied voltage of 4 V. It can be seen from the SEM images that a small amount of white precipitates were attached to the electrode surface under 4 V conditions. These were mainly salts that adhere to the electrode surface by electrostatic adsorption. Compared with the CB electrode, a small amount of Cu, Fe, and Zn appeared in the CM anode, which was mainly due to the strong adsorption property of activated carbon in CM. With the voltage of 4 V, the actual voltage exceeds the decomposition voltage of ions in the solution. The oxidation decomposition of CN^−^ → CNO^−^ → CO_2_ + N_2_, and SCN^−^ mainly occurred and there was a small amount of hydrolysis side effects.

### SEM-EDS Spectrum Analysis of Cathode

**Figure 5:**
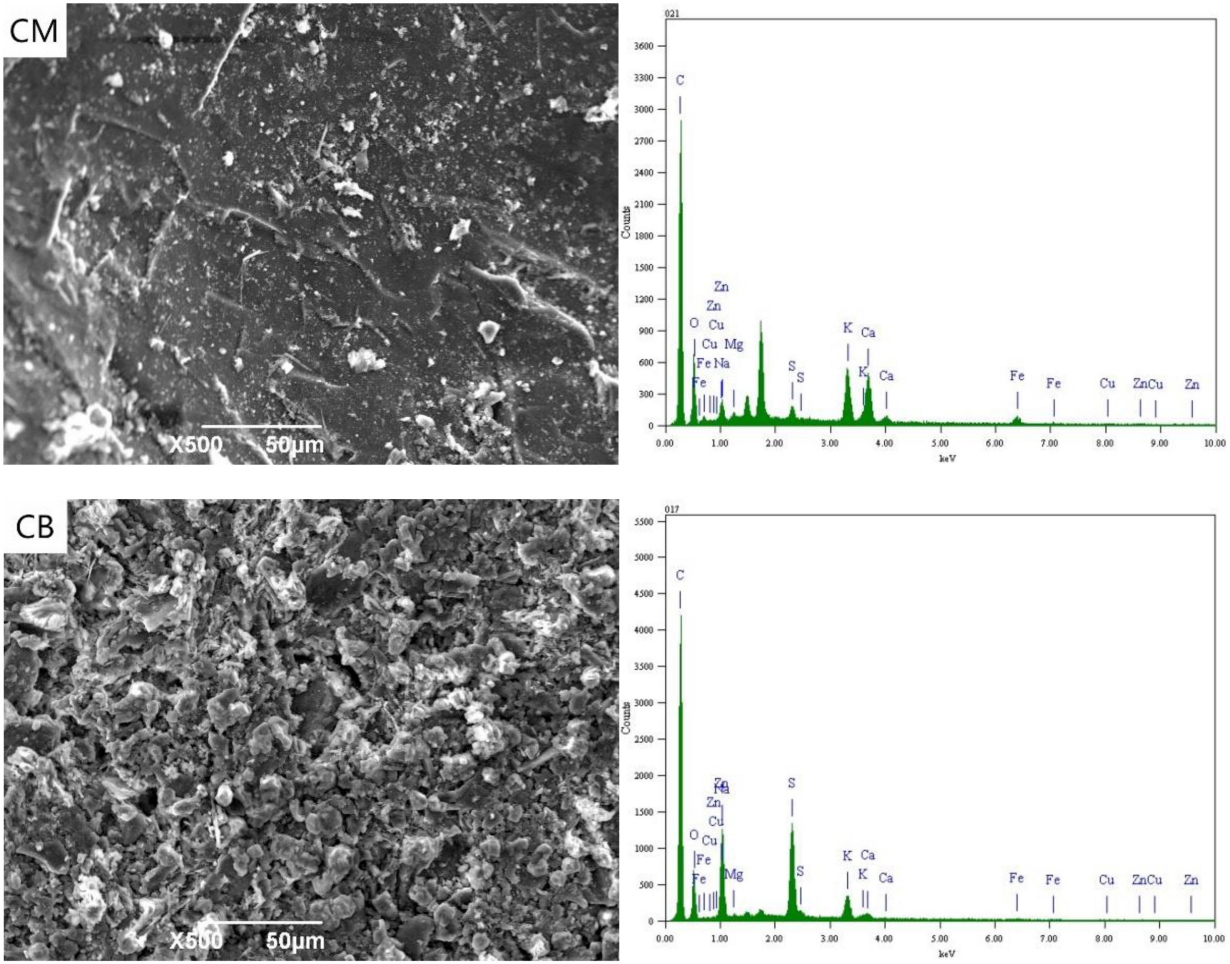
SEM-EDS spectrum of cathode under 2 V at 3D system.

Figure 5 shows the SEM-EDS of CM and CB cathode with a voltage of 2 V. At 2 V, the applied voltage in the solution exceeds the decomposition voltage of water, and a large number of bubbles were generated in the cathode, and a hydrogen evolution reaction occurs. It can be seen from the SEM images that there were a lot of K, Mg, and other metals salts on the surface of CM and CB, which were caused by the cations' directional migration toward the cathode under the electric field. In the energy spectrum of CM, the cathode appears Fe. Due to the Fe in the stainless steel mesh, the complexing reaction with CN^−^ at 2 V takes place, which then adsorbs on the cathode.

**Figure 6:**
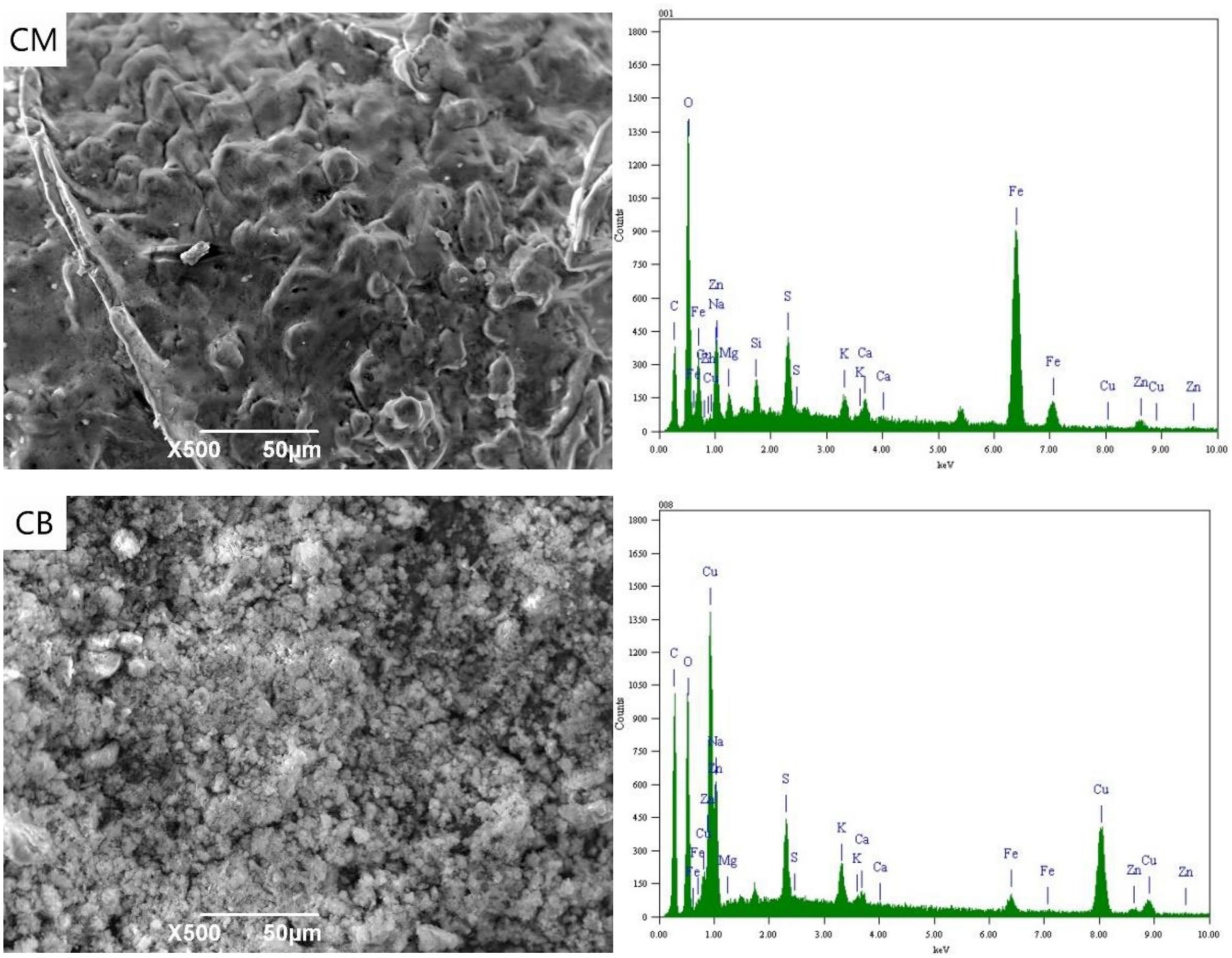
SEM-EDS spectrum of cathode under 4 V at 3D system.

Figure 6 shown the SEM-EDS spectrum of CM and CB electrodes. When the voltage is 4 V, the content of Cu, Zn, and other complex ions in the solution were greatly reduced. According to the energy spectrum, the metal content on the cathode surface was greatly increased, mainly because of the reduction deposition of Cu, Fe, and Zn on the cathode. The results show that the content of Fe in CM system was higher than that in CB, that may the stainless steel mesh dissolves in solution, by CN^−^ and Fe ion generated complex which was deposited on the cathode at 4 V. However, in the spectrum of the CB electrode, the higher Cu content was mainly based on electrodeposition, while the Cu content in CM was relatively low. On the one hand, only a small part of activated carbon in the cathode was used for detection and analysis, on the other hand, this depends on the resistance and the conduction efficiency of CM.

### SEM-EDS Spectrum Analysis of Particle Electrode

**Figure 7:**
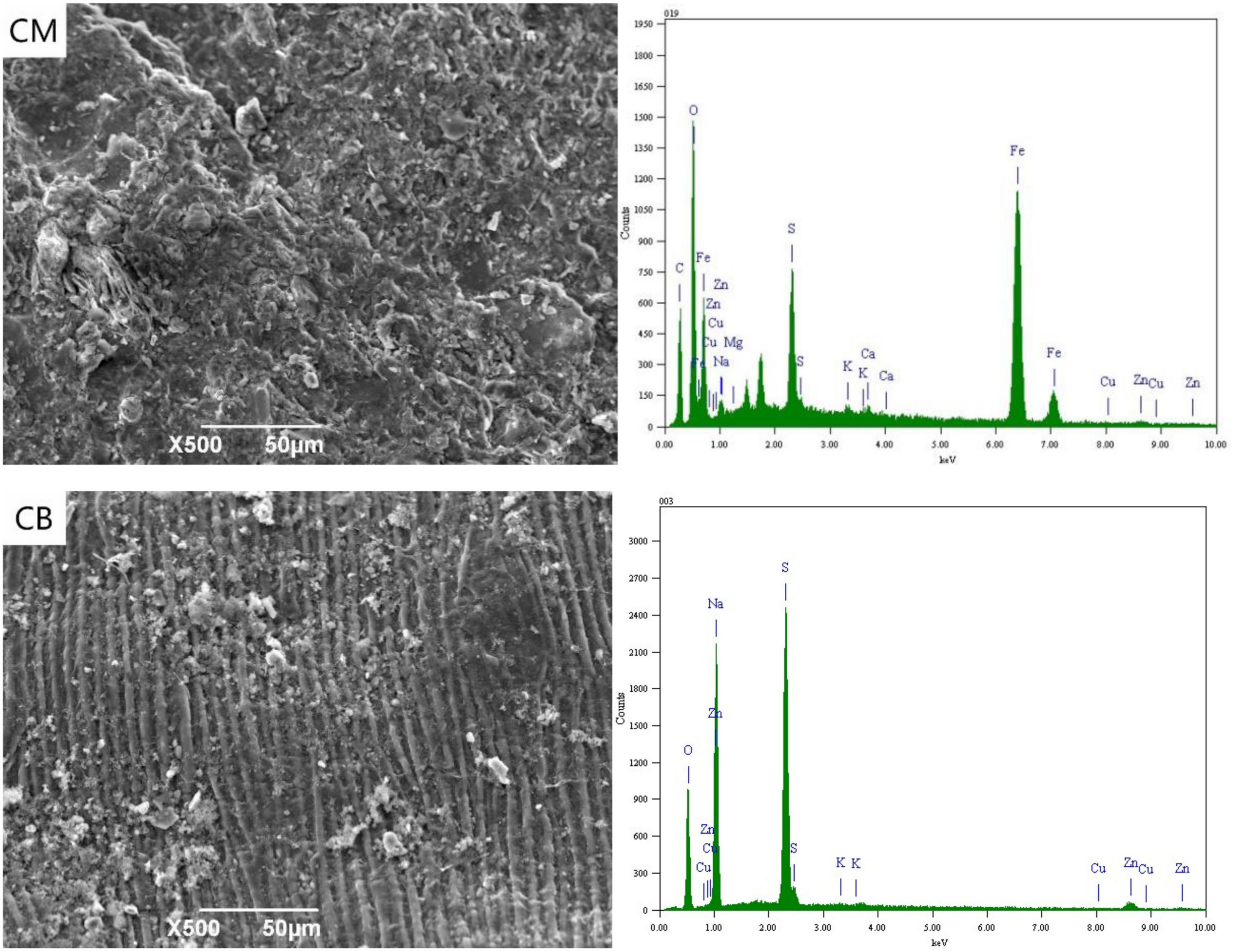
SEM-EDS spectrum of particle electrode under 2 V at 3D system.

Figure 7 shows the SEM-EDS spectra of CM and CB at 2 V. The analysis samples were a small part of the experimental particle activated carbon. With the applied voltage of 2 V, the energy spectrum indicates that there were a lot of metal ions on the surface of the particle electrode under the action of electric adsorption. The metal complex adsorption on activated carbon in the ordered ${\text{Fe(CN)}}_{6}^{4-}$, ${\text{Zn(CN)}}_{4}^{2-}$, and ${\text{Cu(CN)}}_{3}^{2-}$ (Yonghui et al., 2017b). The ferricyanide produced by the complexation with stainless steel mesh and from wastewater will be adsorbed on the activated carbon at first, so the particle electrode surface of CM as the main electrode has a higher content of Fe ion. At 2 V, the action mechanism of the particle electrodes was electric adsorption and adsorption. Magnetic stirring would lead to the bipolar of the particle electrode, which would enhance the efficiency of electric adsorption and the removal rate of ions.

**Figure 8:**
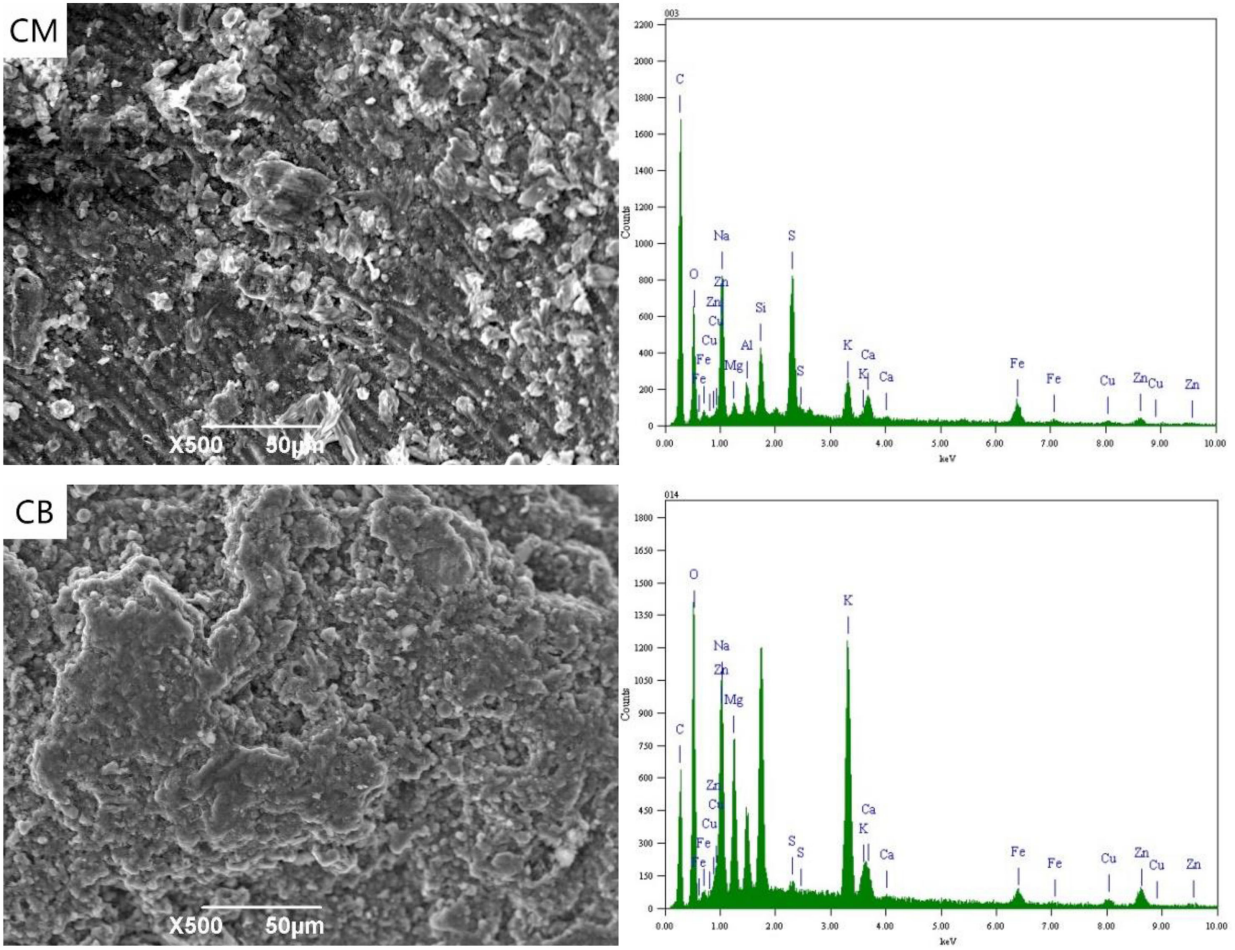
SEM-EDS spectrum of particle electrode under 4 V at 3D system.

Figure 8 shows the SEM-EDS spectra of the particle electrodes at 4 V. As an important part of the 3D system, particle electrodes play the important roles of electron transfer and mass-transfer, acting as the site of an electrochemical reaction. At 4 V, the particle electrodes were considered as micro electrolysis cells. The energy spectrum indicates that there were a certain amount of metal elements present, such as Fe, Cu, and Zn on the surface of the electrode, while the contents of Cu and Zn in the particle electrodes of CB were higher than that of CM. This is mainly because the resistance and conduction efficiency of CM reduces the voltage between the particle electrodes and hinders the electrodeposition reaction of partial Cu and Zn.

### XRD Analysis of the Precipitate

**Figure 9:**
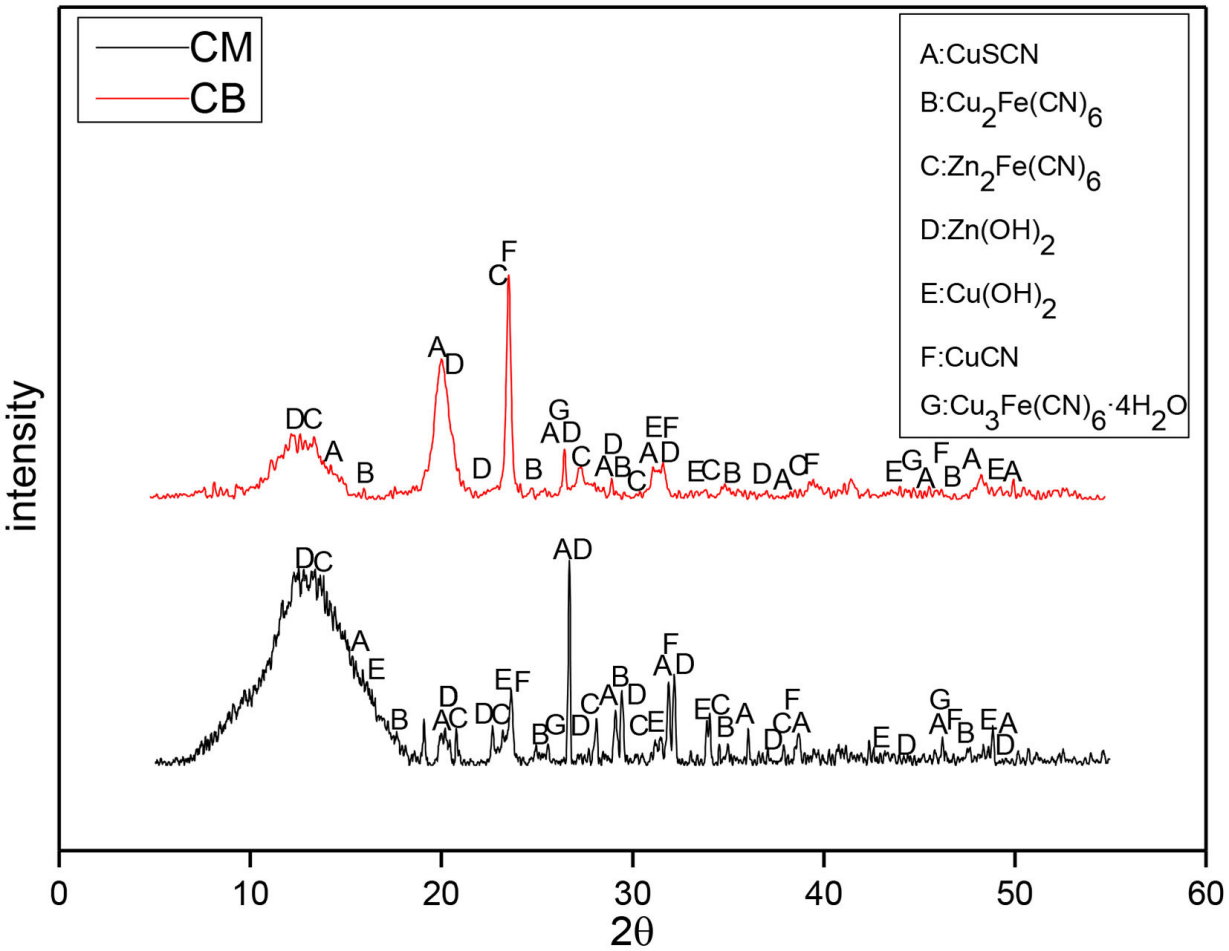
XRD of precipitates of the different electrodes under 2 V at 3D system.

To study the reaction mechanism furtherly, the precipitates in the solution under different electrode systems and voltages were analyzed by XRD. The XRD analysis results of the precipitates at 2 V are shown in Figure 9. At 2 V, the main components of the precipitates between the activated carbon filled stainless steel mesh (CM) and coal based electrode (CB) were similar, and the main precipitates were CuSCN, Zn_2_Fe(CN)_6_, Zn(OH)_2_, and CuCN. The difference is that the precipitate contains a large amount of Zn_2_Fe(CN)_6_ when the electrode was CM. This is because the iron in the stainless steel mesh would be dissolved in the solution and combine with the cyanide ion to form a large number of iron cyanide complexes, and precipitate with zinc in the solution to generate a large amount of Zn_2_Fe(CN)_6_.

**Figure 10:**
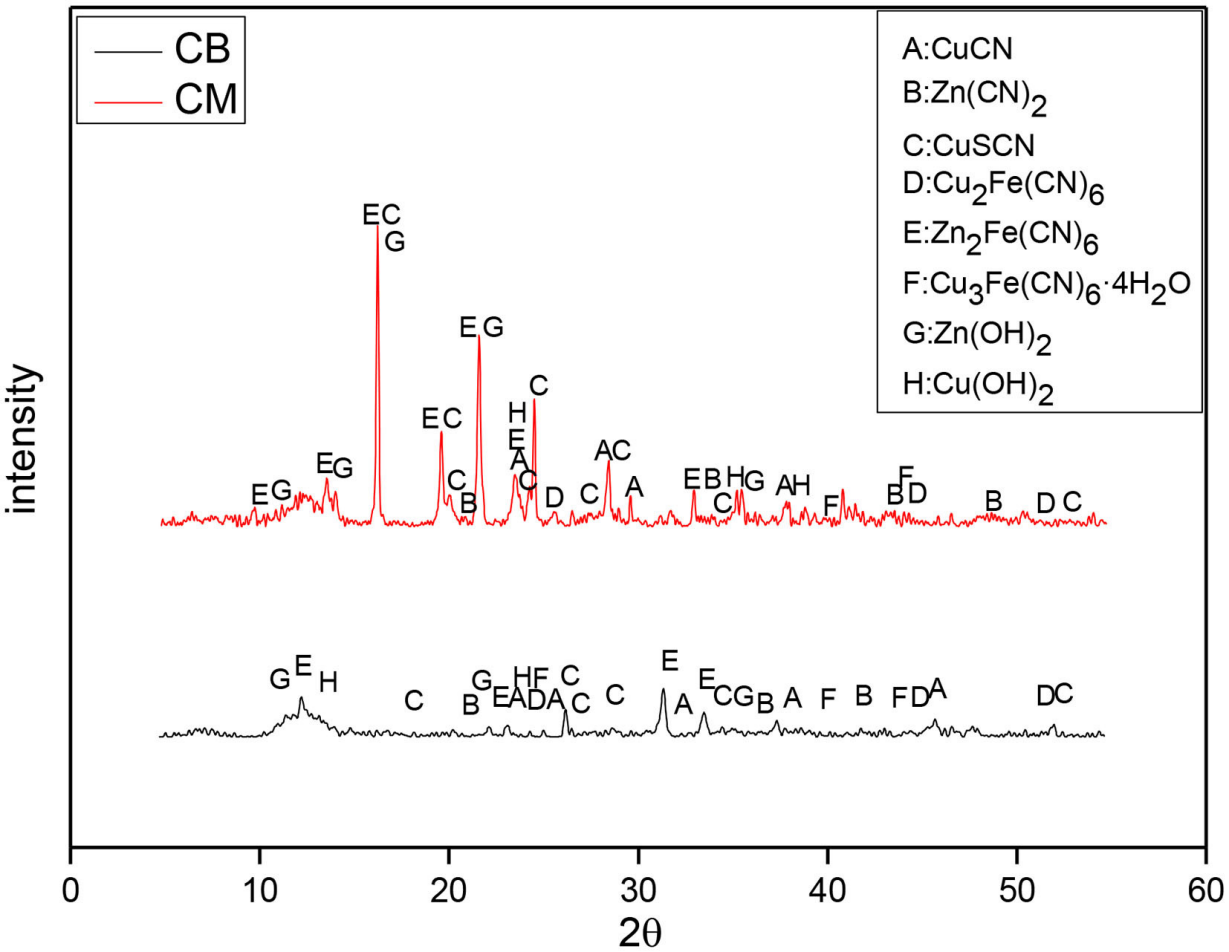
XRD of precipitates of the different electrodes under 4 V at 3D system.

The XRD of the precipitates at 4 V is shown in Figure 10, which indicates that the content of Zn_2_Fe(CN)_6_ in the precipitates of the CM system was high. This is mainly caused by the Fe dissolved in the solution. The precipitation of the CM system was much larger than that of the CB electrode, which proves that under the condition of 4 V, due to the influence of the CM material's resistance and conduction efficiency, the actual voltage in the solution system may not reach the deposition voltage of Cu and Zn. It could also be because only a small amount of metal separates, so the precipitation reaction of Cu and Zn mainly occurs in the solution. However, the electrodeposition reaction of metal mainly occurs in the CB electrode system, the quantity of precipitate was smaller.

### The Mechanism Analysis of 3D System

**Figure 11:**
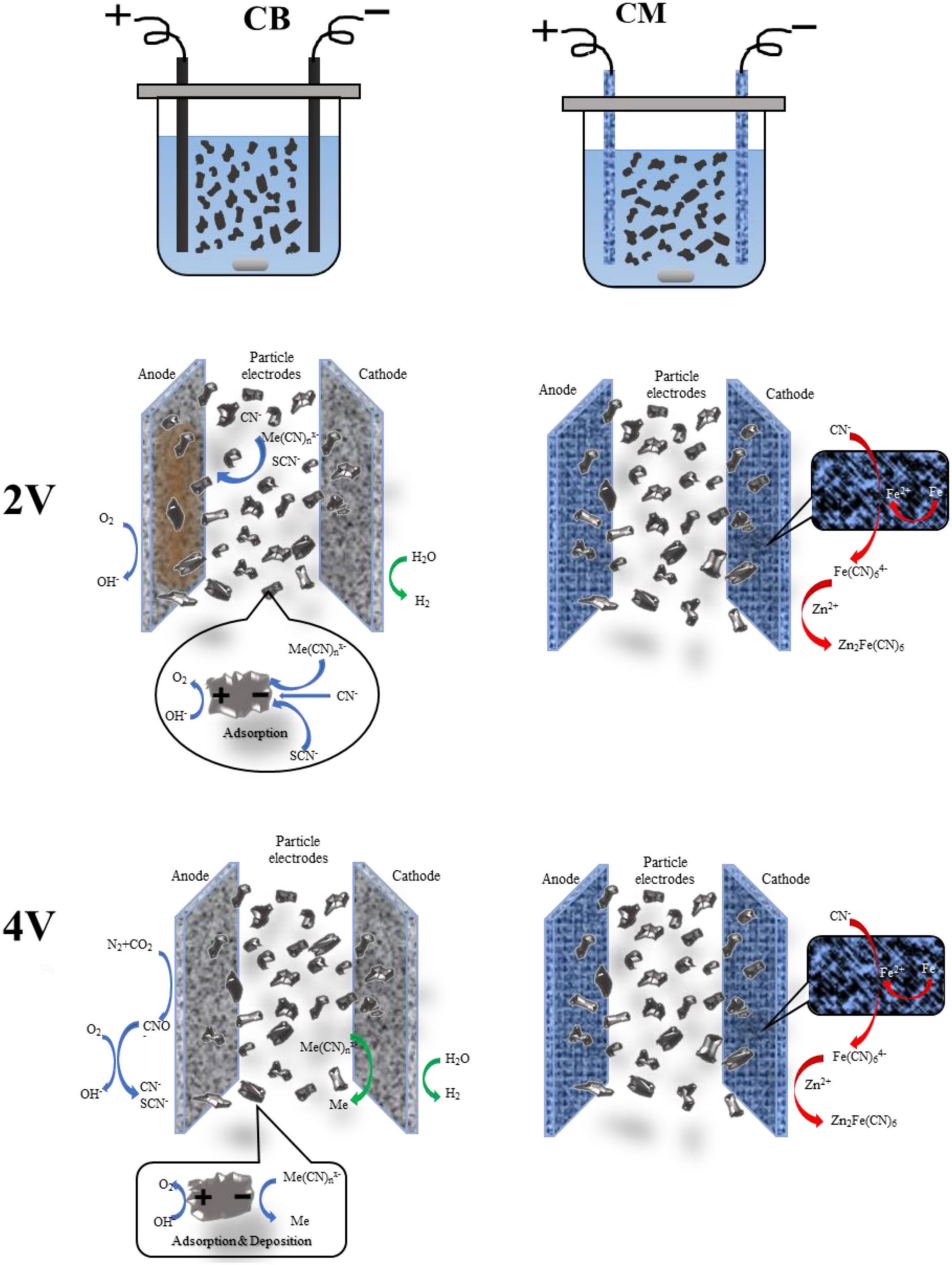
Schematic diagram of the mechanism under different voltages. (1) Me-Cu, Zn, Fe. (2) The reaction of CM were as same as CB, except for special marked.

According to the research results above, which used CM and CB as an anode and cathode, and granular activated carbon (GAC) as particle electrode, when cyanide wastewater was treated by the 3D system under different voltage conditions, the ions in the solution interacted by adsorption, directional migration, enrichment precipitation, and electrolytic deposition. Under the action of different voltages, the reactions in the solution were different. The main mechanism is shown in Figure 11.

At a voltage of 2 V, the actual voltage in the system exceeds the decomposition voltage of water, which produced O_2_ in the anode, and caused a large amount of H^+^ to gather near the anode, and complex precipitation reaction (Yonghui et al., 2017b) occurred, mainly forming CuSCN, Cu_2_Fe(CN)_6_, Zn_2_Fe(CN)_6_, and so on. The iron in the CM electrode dissolution also leads to the formation of Zn_2_Fe(CN)_6_ with the iron cyanide complex and zinc ion in the solution. The reaction equations were as follows:

(1) $$\begin{array}{c} 4\text{O}{\text{H}}^{-}\to {\text{O}}_{2}+2{\text{H}}_{2}\text{O}+4\text{e} \end{array}$$

(2) $$\begin{array}{c} 2{\text{H}}^{+}+2{\text{e}}^{-}\to H_{2} \end{array}$$

(3) $$\begin{array}{c} \text{Cu}{\left(\text{CN}\right)}_{3}^{2-}+2{\text{H}}^{+}=\text{CuCN}↓+2\text{HCN} \end{array}$$

(4) $$\begin{array}{c} \text{Zn}{\left(\text{CN}\right)}_{4}^{2-}+2{\text{H}}^{+}=\text{Zn}{\left(\text{CN}\right)}_{2}↓+2\text{HCN} \end{array}$$

(5) $$\begin{array}{c} \text{CuCN}+\text{SC}{\text{N}}^{-}+{\text{H}}^{+}=\text{HCN}↑+\text{CuSCN}↓ \end{array}$$

(6) $$\begin{array}{c} 2\text{C}{\text{u}}^{2+}+\text{Fe}{\left(\text{CN}\right)}_{6}^{4-}=\text{C}{\text{u}}_{2}\text{Fe}{\left(\text{CN}\right)}_{6} \end{array}$$

(7) $$\begin{array}{c} 2\text{Z}{\text{n}}^{2+}+\text{Fe}{\left(\text{CN}\right)}_{6}^{4-}=\text{Z}{\text{n}}_{2}\text{Fe}{\left(\text{CN}\right)}_{6} \end{array}$$

(8) $$\begin{array}{c} 3\text{C}{\text{u}}^{+}+{\left[\text{Fe}{\left(\text{CN}\right)}_{6}\right]}^{3-}+4{\text{H}}_{2}\text{O}=\text{C}{\text{u}}_{3}\text{Fe}{\left(\text{CN}\right)}_{6}\cdot 4{\text{H}}_{2}\text{O} \end{array}$$

(9) $$\begin{array}{c} \text{Z}{\text{n}}^{2+}+2\text{O}{\text{H}}^{-}=\text{Zn}{\left(\text{OH}\right)}_{2} \end{array}$$

(10) $$\begin{array}{c} \text{C}{\text{u}}^{2+}+2\text{O}{\text{H}}^{-}=\text{Cu}{\left(\text{OH}\right)}_{2} \end{array}$$

In the 3D system, the actual voltage at both ends of the electrode was higher than the precipitation voltage of Cu, Zn, and Fe at 4 V (Yonghui et al., 2017a), and electrodeposition occurs on the cathode, i.e., Cu, Zn, and Fe were formed, as shown in formulas (14–17). While on the anode, CN^−^ and SCN^−^ were converted into CNO^−^ firstly, and then CO_2_ and N_2_ were mainly generated. At the same time, water decomposition reaction also exists in the anode and cathode [formulas (1, 2)]. The reaction of the particle electrode is shown in the figure, under the action of the electric field the oxidation-reduction reaction occurs at both ends of the particle electrode (Yonghui et al., 2019). Thus, the 3D system is more efficient for wastewater treatment, and can reach more than 90%.

(11) $$\begin{array}{c} \text{C}{\text{N}}^{-}+2\text{O}{\text{H}}^{-}\to \text{CN}{\text{O}}^{-}+{\text{H}}_{2}\text{O}+2\text{e} \end{array}$$

(12) $$\begin{array}{c} 2\text{CN}{\text{O}}^{-}+4\text{O}{\text{H}}^{-}\to 2\text{C}{\text{O}}_{2}+{\text{N}}_{2}+2{\text{H}}_{2}\text{O}+6\text{e} \end{array}$$

(13) $$\begin{array}{c} \text{SC}{\text{N}}^{-}+8\text{O}{\text{H}}^{-}=\text{S}{\text{O}}_{4}^{2-}+\text{C}{\text{N}}^{-}+4{\text{H}}_{2}\text{O}+6\text{e} \end{array}$$

(14) $$\begin{array}{c} \text{Cu}{\left(\text{CN}\right)}_{4}^{3-}+\text{e}\to \text{Cu}+4\text{C}{\text{N}}^{-}\varphi =-1.292 \end{array}$$

(15) $$\begin{array}{c} \text{Zn}{\left(\text{CN}\right)}_{4}^{2-}+2\text{e}\to \text{Zn}+4\text{C}{\text{N}}^{-}\varphi =-1.385 \end{array}$$

(16) $$\begin{array}{c} \text{Fe}{\left(\text{CN}\right)}_{6}^{3-}+3\text{e}\to \text{Fe}+6\text{C}{\text{N}}^{-}\varphi =-1.042 \end{array}$$

(17) $$\begin{array}{c} \text{Fe}{\left(\text{CN}\right)}_{6}^{4-}+2\text{e}\to \text{Fe}+6\text{C}{\text{N}}^{-}\varphi =-1.742 \end{array}$$

## Conclusion

(1) The results of the present study show that the 3D electrode system with activated carbon filled stainless steel mesh (CM) and coal-based electrode (CB) are better than stainless steel mesh (M) as the main electrode. The CB electrode is especially suitable for cyanide wastewater treatment. The main mechanism of the system of 2 V is electro adsorption and enrichment precipitation. At 4 V, the system mainly uses electrodeposition to remove all ions in wastewater, and the treatment efficiency of electrodeposition was higher than that of electro adsorption.

(2) The treatment efficiency of the 3D system is higher than that of the 2D system because of the addition of the particle electrode the reaction efficiency and removal rate of ions in wastewater improve greatly. When the applied voltage of the 3D system is 4 V, with a treatment time of 5 h, an electrode spacing of 10 mm, and the dosage of activated carbon particles is 2 g, the removal rates of Cu, Zn, CN_T_, CN^−^, and SCN^−^ in wastewater were 94.53, 98.14, 94.14, 98.55, and 93.13%, respectively. The surface of the CB anode has an oxygen evolution reaction and electrochemical oxidation reaction of CN^−^, SCN^−^, and there is electrolytic deposition of Cu, Zn, and other metal ions on the cathode surface. There was adsorption and electro adsorption of various ions on the surface of the activated carbon particle electrode, and the electrolytic deposition reaction of Cu, Zn, and other metal ions at the same time.

(3) In the process of cyanide wastewater treatment by 3D electrode based on CB electrode, due to the concentration of hydrogen ion increase in the local area near the anode caused by oxygen evolution reaction, the precipitation reaction between CN^−^, SCN^−^ and metal cyanide complex ions will occur under the action of the electric field, generating precipitation of CuSCN, Cu_2_Fe(CN)_6_, Zn_2_Fe(CN)_6_, which is the main reason for the removal of cyanide and heavy metals ions from cyanide wastewater.

## Data Availability Statement

The original contributions presented in the study are included in the article/supplementary materials, further inquiries can be directed to the corresponding author.

## Author Contributions

YS: conceptualization, methodology, writing—reviewing, and editing. SL: data curation, writing—original draft, software, and formal analysis. Both authors: contributed to the article and approved the submitted version.

## Conflict of Interest

The authors declare that the research was conducted in the absence of any commercial or financial relationships that could be construed as a potential conflict of interest.
